# Cassava brown streak disease: historical timeline, current knowledge and future prospects

**DOI:** 10.1111/mpp.12613

**Published:** 2017-12-28

**Authors:** Katie R. Tomlinson, Andy M. Bailey, Titus Alicai, Sue Seal, Gary D. Foster

**Affiliations:** ^1^ School of Biological Sciences University of Bristol Bristol BS8 1TQ UK; ^2^ National Crops Resources Research Institute Kampala 7084 Uganda; ^3^ Natural Resources Institute University of Greenwich Chatham Maritime Kent ME4 4TB UK

**Keywords:** CBSD, CBSV, CMD, food security, UCBSV, virology

## Abstract

Cassava is the second most important staple food crop in terms of per capita calories consumed in Africa and holds potential for climate change adaptation. Unfortunately, productivity in East and Central Africa is severely constrained by two viral diseases: cassava mosaic disease (CMD) and cassava brown streak disease (CBSD). CBSD was first reported in 1936 from northeast Tanzania. For approximately 70 years, CBSD was restricted to coastal East Africa and so had a relatively low impact on food security compared with CMD. However, at the turn of the 21st century, CBSD re‐emerged further inland, in areas around Lake Victoria, and it has since spread through many East and Central African countries, causing high yield losses and jeopardizing the food security of subsistence farmers. This recent re‐emergence has attracted intense scientific interest, with studies shedding light on CBSD viral epidemiology, sequence diversity, host interactions and potential sources of resistance within the cassava genome. This review reflects on 80 years of CBSD research history (1936–2016) with a timeline of key events. We provide insights into current CBSD knowledge, management efforts and future prospects for improved understanding needed to underpin effective control and mitigation of impacts on food security.

## Introduction

Cassava (*Manihot esculenta* Crantz, family Euphorbiaceae) produces carbohydrate‐rich storage roots, which are a staple food crop for approximately 800 million people worldwide [Food and Agriculture Organization (FAO), 2013]. In Africa, cassava is the second most important food staple in terms of per capita calories consumed (Nweke, 2004). Storage roots are used as a fresh carbohydrate source and can also be processed into flour, which may be consumed by the grower's family, sold in local markets or used to produce several industrial food products (Hillocks and Thresh, 2002). Subsistence farmers rely on cassava for a vital energy source, as it can be planted and harvested throughout the year, tolerates periods of unpredictable drought and grows on marginal soils (Hillocks and Thresh, 2002). Recent modelling has suggested that cassava may be highly resilient to future climate change and could provide Africa with adaptation opportunities, which are not offered by other staple food crops (Jarvis *et al*., 2012).

Cassava was introduced into Africa from Brazil by Portuguese traders in the 16th century and subsequently integrated into local agriculture in countries across the continent (Jones, 1959). Africa produces over one‐half of global cassava (57%) (Bennett, 2015); however, the continent's average fresh yield (9.9 tonnes/ha) lags behind potential yields (15–40 tonnes/ha) achieved under experimental conditions (Fermont *et al*., 2009). There are many reasons behind the reduced yields, including restricted access to labour, poor soil quality and premature harvesting (Fermont *et al*., 2009). Productivity in East and Central Africa is significantly constrained by two viral diseases, cassava mosaic disease (CMD) and cassava brown streak disease (CBSD), which together are estimated to cause annual losses worth US$1 billion [International Institute of Tropical Agriculture (IITA), 2014a] and adversely affect food security in the entire region (Patil *et al*., 2015).

**Figure 1:**
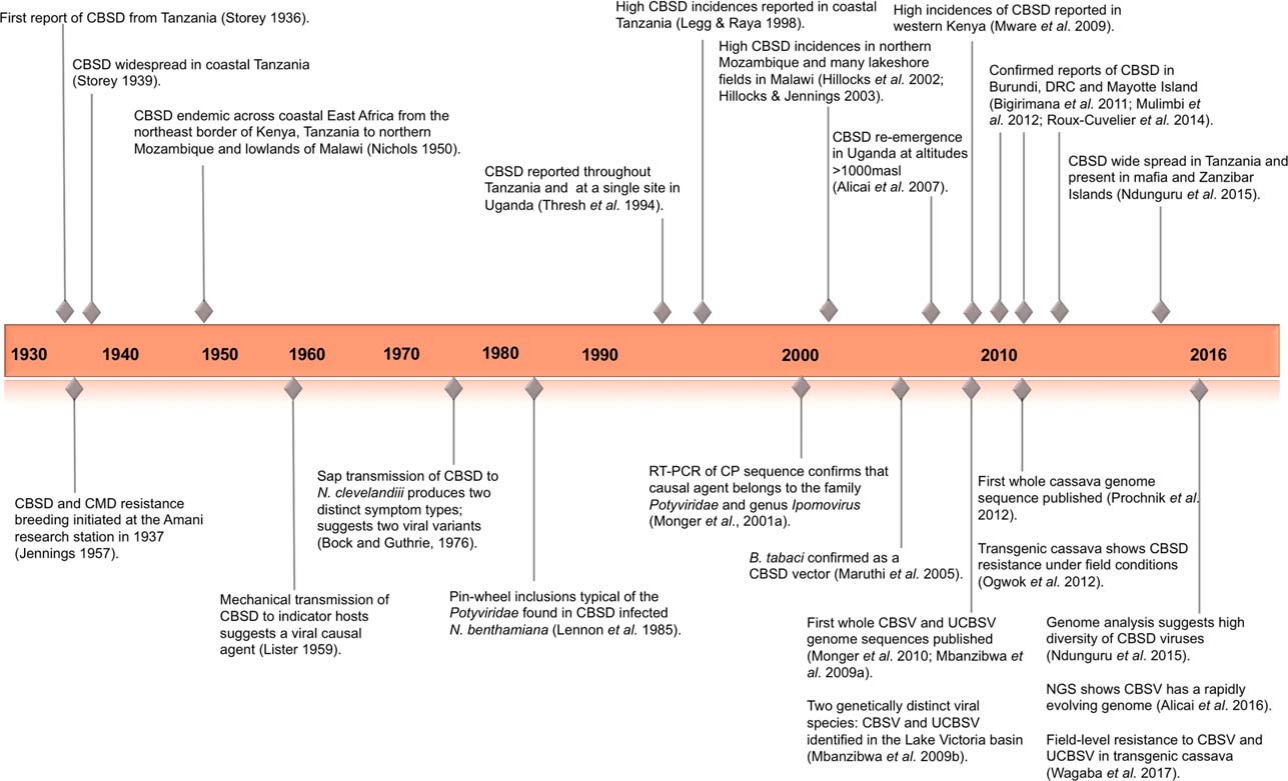
Key events in cassava brown streak disease (CBSD) geographical distribution and research history (1936–2016). CBSV, *Cassava brown streak virus*; CMD, cassava mosaic disease; NGS, next‐generation high‐throughput sequencing; RT‐PCR, reverse transcription polymerase chain reaction; UCBSV, *Ugandan cassava brown streak virus*.

**Figure 2:**
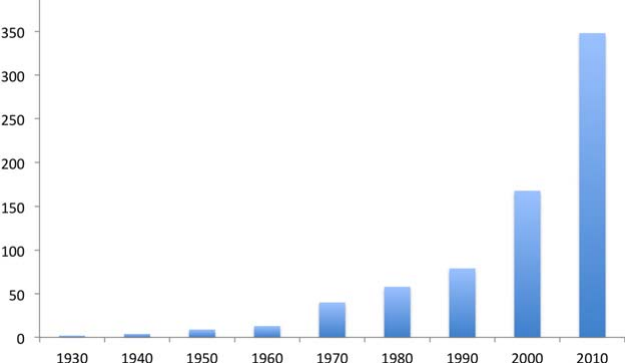
Cumulative number of scientific papers, reports or reviews which refer to cassava brown streak disease (CBSD), published in each decade between 1936 and 2016; dramatic increase in publications from the mid‐1990s following the re‐emergence of CBSD (accessed on Google scholar in December 2016).

In this article, we review CBSD research history, highlighting key events in a timeline (Fig. 1), and provide future prospects for further understanding and effective control. The review is split into two phases according to the geographical distribution of CBSD. The first phase covers the small number (*n* = 65) of reports published between 1936 and the early 1990s when CBSD was reported to be restricted to low‐altitude areas [<1000 m above sea level (masl)] along coastal East Africa and lakeshore districts of Malawi (Fig. 2) (Legg *et al*., 2011). The second phase examines CBSD re‐emergence after the mid‐1990s, when CBSD spread across East and Central Africa (Legg *et al*., 2011). We review the corresponding increased number (*n* = 277) of reports on CBSD geographical expansion, viral molecular characterization, host interactions, diagnostic techniques and control efforts.

We offer insights into what can be learnt from CBSD history, in particular the need for the application of knowledge to protect against and predict multiple biotic threats to staple food crops through improved understanding of CBSD epidemiology, diagnostics, surveillance and predictive modelling. This calls for effective international scientific collaborations across multiple areas of expertise and the rapid application of research and technologies to solve problems affecting farmers.

## Initial Emergence and Symptom Description (1930s to the Early 1990s)

**Figure 3:**
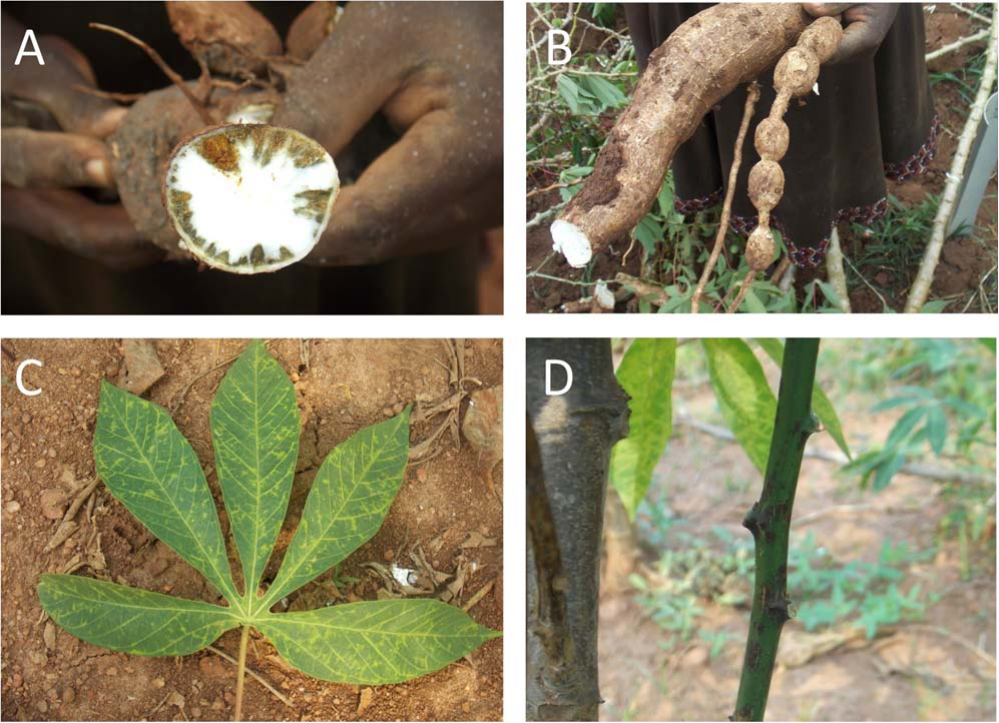
Cassava brown streak disease (CBSD) storage root necrosis (A), radial root constrictions (B), foliar chlorosis (C) and brown streaks or lesions on stems (D).

The first report of CBSD from northeast Tanzania (then called Tanganyika) describes distinctive foliar symptoms on lower mature cassava leaves and rot of storage roots (Storey, 1936). Nichols (1950) later reported that symptoms could be expressed on all parts of the plant and could include storage root necrosis (Fig. 3A), radial root constrictions (Fig. 3B), foliar chlorosis (Fig. 3C) and, occasionally, brown streaks or lesions on stems (Fig. 3D).

It was noted that two main types of foliar symptoms exist: (i) feathery chlorosis along secondary vein margins, which eventually coalesce to form blotches, and (ii) chlorotic mottling with no veinal association (Nichols, 1950). These distinctive symptoms lack the leaf distortion observed in CMD‐infected cassava plants. CBSD symptoms are variable in terms of severity, onset of symptom expression and parts of the plant affected, depending on the viral strain, cassava cultivar, environmental conditions and age of the plant when infected (Nichols, 1950). This variability makes diagnosis difficult for farmers (Nichols, 1950) and can result in farmers being unaware that their crop is affected until they harvest storage roots (Legg and Kanju, 2015). The difficulty in the diagnosis of CBSD has meant that infected stems have been transported to areas in which CBSD has previously been absent and used for planting material. Symptom variability has also hampered epidemiological studies, as the disease can go unnoticed in an area for long periods.

The surveying and symptom scoring of infected plants across different geographical areas have revealed that most plants with foliar symptoms usually also develop root necrosis (Hillocks *et al*., 2001). In the most sensitive cultivars, CBSD reduces root weight by up to 70% with necrosis developing at 6 months post‐planting (Hillocks *et al*., 2001). However, it has been reported that the local Tanzanian cultivar ‘Nachinyaya’ does not develop root necrosis and therefore is relatively tolerant to CBSD (Hillocks *et al*., 1996).

## Early Geographical Distribution

Storey (1939) reported that CBSD was widespread in coastal Tanzania and, by 1950, the disease was endemic across coastal areas of East Africa from northeast Kenya, Tanzania to northern Mozambique at altitudes below 1000 masl (Nichols, 1950). The disease was reported in Uganda in 1945 and may have been introduced through infected cuttings sent from the Amani research station in Tanzania (Jameson, 1964; Nichols, 1950). Strict roguing of infected plants, replacement with non‐infected planting material and quarantine appear to have prevented the spread of CBSD in Uganda at this time (Nichols, 1950). Significantly, a lack of plant‐to‐plant vector transmission at higher altitudes was reported (Jennings, 1960; Nichols, 1950).

## Causal Agent Characterization

Storey (1936) suspected a viral causal agent, as CBSD was successfully transmitted through the grafting of stem cuttings. Subsequently, Lister (1959) mechanically transmitted CBSD to indicator hosts, including *Petunia hybrida*, *Datura stramonium*, *Nicotiana tabacum*, *N. rustica* and *N. glutinosa*, which produce a range of symptoms depending on the sensitivity of the host and viral variant. In 1976, sap transmission of CBSD from infected cassava material to *N. clevelandii* produced two distinct symptom types, which suggested that two viral variants may be responsible for CBSD (Bock and Guthrie, 1976).

Virus particles were identified by electron microscopy analysis of CBSD‐infected *N. debneyi* (Bock, 1994). The infected samples contained 650‐nm filamentous particles with a similar morphology to viruses within the *Carlavirus* genus (Bock, 1994). However, pinwheel inclusions, typical of *Potyviridae*, were identified in CBSD‐infected *N. benthamiana* (Lennon *et al*., 1985). Pinwheel inclusions were subsequently found through more thorough electron microscopy of CBSD‐infected cassava samples, albeit at low concentrations (Were *et al*., 2004).

The *Potyviridae* sequence identity was finally confirmed in 2001 through reverse transcription‐polymerase chain reaction (RT‐PCR) of CBSD‐infected *N. benthamiana* samples (Monger *et al*., 2001a). When the RT‐PCR product was sequenced, it aligned most closely to the coat protein sequence of *Sweet potato mild mottle virus* (SPMMV, genus *Ipomovirus*, family *Potyviridae*) (Monger *et al*., 2001a). The same RT‐PCR technique was used to detect *Cassava brown streak virus* (CBSV) in symptomless cassava leaves, highlighting the sensitivity of the RT‐PCR technique (Monger *et al*., 2001a).

## Early Control Efforts

In the 1930s, a cassava breeding programme was launched in Tanzania, which included breeding for CBSD and CMD resistance at the Amani research station (Jennings, 1957; Nichols, 1946). Early breeding to develop virus‐resistant cultivars involved crossing cultivated cassava with wild relatives, including *M. glaziovii*, *M. dichotoma*, *M. catingea*, *M. saxicola* and *M. melanobasis*, which are believed to possess higher levels of CBSD resistance (Jennings, 1957; Kawuki *et al*., 2016). The breeding programme produced the *M. esculenta*–*M. glaziovii* hybrid, known as ‘Namikonga’ in Tanzania or ‘Kaleso’ in Kenya, which, for many years, offered relatively high levels of CBSD tolerance (Hillocks and Jennings, 2003; Kaweesi *et al*., 2014). However, ‘Namikonga’ was not widely distributed to farmers, which may be because of its susceptibility to CMD (Hillocks and Jennings, 2003; Kawuki *et al*., 2016).

## Initial Vector Transmission Studies

Until relatively recently, very little was known about the vector transmission of CBSVs. It had been noted that CBSD outbreaks tended to coincide with increases in whitefly populations (Hillocks and Jennings, 2003; Storey, 1939). However, initial attempts to transmit CBSV with whitefly (*Bemisia tabaci*) or aphid (*Myzus persicae*) were unsuccessful (Bock, 1994).

## Geographical Distribution in the Early 1990s

In the early 1990s, there were reports of high CBSD incidences in areas of Tanzania, Mozambique and Malawi (Hillocks and Jennings, 2003). Surveys revealed CBSD incidences reaching 36%–50% in cassava fields along coastal areas of Tanzania (Hillocks *et al*., 1999; Legg and Raya, 1998). Similarly, CBSD incidences in Malawi reached 75% in many fields surrounding Lake Malawi and nearly all plants inspected in northern coastal areas of Mozambique expressed CBSD symptoms (Hillocks and Jennings, 2003; Hillocks *et al*., 2002). In a control effort, virus‐free CBSD‐tolerant cultivars were distributed to farmers in Mozambique who depended heavily on CBSD‐sensitive cassava cultivars for food security (Hillocks and Jennings, 2003). CBSD was also re‐discovered in Uganda in 1994 at a site near Entebbe (Thresh *et al*., 1994). This led researchers to call for concerted efforts to understand CBSD through improved surveillance (Hillocks and Jennings, 2003).

## Reflections on Initial Emergence (1930s to Early 1990s)

Despite CBSD being endemic across coastal East Africa during this period, relatively little work was done to understand and control CBSD. This is reflected by the small number of scientific papers, reports or reviews which feature CBSD published between 1936 and the early 1990s (*n* = 65) (Fig. 2). The slight increase in references to CBSD in the 1970s is a result of a small number of reports (*n* = 27) on the threat posed by CBSD. In hindsight, these reports should have served as a warning to take control actions, which may have prevented the later expansion of CBSD across the region.

There was a general lack of scientific interest in CBSD at this time as a result of many factors, including the restricted occurrence of CBSD to low‐altitude areas along coastal eastern Africa and the devastating impacts of CMD on food security. During this period, CMD was a greater priority because of its prevalence across all cassava‐growing areas of Africa, resulting in famines, higher economic losses and forcing many farmers to abandon the crop (Alabi *et al*., 2011; Thresh and Cooter, 2005; Thresh *et al*., 1994). To help control the disease, CMD‐resistant cultivars were distributed to areas severely affected (Legg and Thresh, 2000). Unfortunately, these cultivars showed varying levels of CBSD susceptibility (Legg *et al*., 2006). It is not known whether the deployment of these cultivars contributed to the increased distribution of CBSD in the field.

## Re‐emergence and Expansion Across East and Central Africa (MID‐1990s to 2016)

In 2004, the apparent restriction of CBSD to coastal lowlands changed with the re‐emergence of CBSD at altitudes above 1000 masl (Alicai *et al*., 2007). Infections of cassava plants showing CBSD symptoms at higher altitudes in Uganda were confirmed by RT‐PCR. Coat protein sequences aligned to CBSV isolates from Mozambique and Tanzania with sequence identities from 77.0% to 82.9% (Alicai *et al*., 2007). It is not known whether CBSD had been re‐introduced to Uganda through infected cuttings or whether the disease had existed at a low level since it was first introduced in the 1940s (Alicai *et al*., 2007). Shortly after this first report, the overall incidence of CBSD in Uganda increased from 12% in 2008 to 27% in 2011 (T. Alicai, year of personal communication, 2017), and similar increases were reported in Tanzania and Kenya (Legg *et al*., 2011; Mware *et al*., 2009; Ntawuruhunga and Legg, 2007). There have since been CBSD reports from Burundi (Bigirimana *et al*., 2011), Rwanda (FAO, 2011), eastern Democratic Republic of Congo (Mulimbi *et al*., 2012), south Sudan (T. Alicai, year of personal communication, 2017) and Mayotte Island (Roux‐Cuvelier *et al*., 2014).

It is difficult to obtain a truly accurate estimation of the economic damage caused by CBSD; however, an overall loss of US$750 million a year has been estimated across Kenya, Tanzania, Uganda and Malawi (Hillocks and Maruthi, 2015). CBSD is now one of the leading causes of cassava losses in East Africa (Pennisi, 2010) and its ongoing spread threatens the major cassava‐growing areas of Central and West Africa (Legg *et al*., 2014).

The dramatic increase in the impact of CBSD on food security is reflected in the increase in the number of papers, reports and reviews which refer to CBSD published from the mid‐1990s to 2016 (*n* = 277) (Fig. 2). The expansion of the CBSD epidemic across the Great Lakes region of East and Central Africa has necessitated the rapid development and implementation of effective control strategies. Several important projects were initiated following CBSD re‐emergence, which aimed to develop research, extension and policy capacity in the countries affected. Key targets have been to breed or genetically engineer resistant cultivars, provide certified virus‐clean planting material and improve viral surveillance and diagnosis (Legg *et al*., 2014).

## Recent Local and Regional CBSD Epidemiology

The reasons behind the sudden increase in CBSD incidence and geographical range remain poorly understood. Studies have shown that CBSD spread and development are enhanced by high disease pressure, the use of susceptible genotypes and high whitefly numbers (Katono *et al*., 2015). CBSD is dispersed locally and over long distances through the trade transportation of infected planting material, whereas whiteflies are only able to disperse and amplify CBSD locally (McQuaid *et al*., 2017).

**Figure 4:**
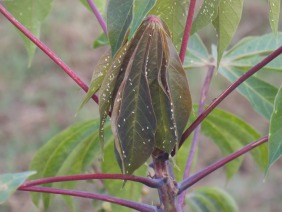
Super‐abundant whiteflies on cassava in Uganda.

The ability of *B. tabaci* to transmit CBSV from infected to healthy plants was confirmed under quarantine insectary and glasshouse conditions by Maruthi *et al*. (2005). It has since been shown that CBSD viruses are transmitted semi‐persistently, with whiteflies acquiring viruses in 5–10 min, retaining them for up to 48 h and transmitting them over relatively short distances of less than 17 m in a cropping season (Maruthi *et al*., 2017). CBSD outbreaks occur from 3 to 12 years after increases in whitefly numbers (Legg *et al*., 2011). Critically, one of the primary causes of the increases in both CMD and CBSD in the African Great Lakes region appears to be super‐abundant numbers of whiteflies (Fig. 4), which are able to thrive at altitudes above 1000 masl (Alicai *et al*., 2007; Jeremiah *et al*., 2015).

Survey data have revealed that the transportation of infected material to areas in which CBSD was previously absent has enabled the disease to spread from independent hot spots (Legg *et al*., 2011). This is because cassava stems used for vegetative planting material are exchanged by farmers across localities and transported over long distances. One report has concluded that plants can also be infected through the use of contaminated cutting tools, which could contribute to in‐field spread (Rwegasira and Chrissie, 2015); however, a similar study has shown that such practices do not result in the transmission of CBSVs (Maruthi *et al*., 2016).

CBSVs are found only in Africa and therefore it appears that these viruses evolved within East Africa on an unknown species and subsequently jumped host into cassava in a new encounter situation (Monger *et al*., 2010). Therefore, there may be other hosts for CBSVs, which could serve as viral inoculum sources in the field (Monger *et al*., 2010). CBSV has been detected in the wild perennial species *M. glaziovii* (Mbanzibwa *et al*., 2011b); the importance of this to CBSD epidemiology is not currently known.

## Molecular Characterization of Unusual CBSVs Genome Features

**Figure 5:**
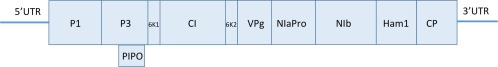
*Cassava brown streak virus* (CBSV) and *Ugandan cassava brown streak virus* (UCBSV) genomes encode a large polyprotein which is autocatalytically cleaved at specific cleavage sequences by virus‐encoded proteases into 10 mature proteins with an additional P3N‐PIPO protein produced through a +2 ribosomal frameshift in the P3 region (Valli *et al*., 2015). CBSV proteins: P1, serine protease/silencing suppressor; P3, third protein; PIPO, pretty interesting Potyviridae open reading frame (ORF), 6K1 and 6K2, 6‐kDa proteins; CI, cylindrical inclusion protein; VPg, viral genome‐linked protein; NIaPro, main viral protease; NIb, viral RNA‐dependent RNA polymerase; Ham1, putative pyrophosphatase; CP, coat protein; UTR, untranslated region. Note unusual features: presence of single P1 protein, absence of helper‐component proteinase protein (HC‐Pro) and presence of novel Ham1 protein (Mbanzibwa *et al*., 2009a).

CBSVs belong to the *Ipomovirus* genus of the *Potyviridae* family (Monger *et al*., 2001a). Ipomoviruses have positive‐sense, single‐stranded genomes, which are translated as large polyproteins and autocatalytically cleaved by virus‐encoded proteases into 10 mature proteins with an additional P3N‐PIPO (N terminal of protein three fused with the Pretty Interesting Potyviridae ORF) protein produced through ribosomal frameshifting (Valli *et al*., 2015). The genomic organization of CBSVs is shown in Fig. 5.

The molecular characterization of coat protein sequences has revealed that there are at least two genetically distinct species: *Cassava brown streak virus* (CBSV) and *Ugandan cassava brown streak virus* (UCBSV) (Monger *et al*., 2001b; Winter *et al*., 2010), which typically have 76%–78% nucleotide and 87%–90% amino acid identity (Mbanzibwa *et al*., 2009b).

Genomic analysis has revealed that CBSVs share unusual features (Mbanzibwa *et al*., 2009a; Monger *et al*., 2010). First, CBSVs lack the multifunctional helper‐component proteinase protein (HC‐Pro), which possesses silencing suppressor, vector transmission and long‐distance movement *in planta* activities in *Potyviridae* viruses (Valli *et al*., 2015). The HC‐Pro protein is found in all other known *Potyviridae* viruses, except for *Squash vein yellowing virus* (SqVYV) and *Cucumber vein yellowing virus* (CVYV) (Mbanzibwa *et al*., 2009a). In CBSVs, HC‐Pro appears to have been replaced by silencing suppressor activity of the P1 serine proteinase (Mbanzibwa *et al*., 2009a). CBSV and UCBSV P1 proteins are most closely related to P1 of SPMMV and P1b of SqVYV and CVYV, which are related to the tritomovirus P1 proteins (Mbanzibwa *et al*., 2009a). The CBSV and UCBSV P1 proteins both contain zinc finger and LXKA motifs (Mbanzibwa *et al*., 2009a). The zinc finger and LXKA motifs in CVYV P1b are essential for silencing suppressor activity through the binding and sequestering of small interfering RNA (siRNA) required for silencing (Valli *et al*., 2008). It is therefore likely that the same motifs are responsible for the silencing suppressor activities of CBSV and UCBSV P1 proteins (Mbanzibwa *et al*., 2009a).

CBSV and UCBSV also encode novel Ham1 proteins with conserved Maf/Ham1 motifs (Mbanzibwa *et al*., 2009a). Proteins with Maf/Ham1 domains are found across prokaryotic and eukaryotic organisms and have nucleoside triphosphate pyrophosphatase activities, which reduce mutation rates by preventing the incorporation of non‐canonical nucleotides into RNA and DNA (Galperin *et al*., 2006). The functions of CBSV and UCBSV Ham1 proteins are yet to be elucidated, but they are likely to provide essential functions in the lifecycles of CBSVs. For instance, Ham1 proteins may reduce mutation rates under oxidative stress conditions in mature cassava leaves where CBSV viruses are found at the highest concentrations within the plant (Ogwok *et al*., 2014). *Euphorbia ring spot virus* (EuRV, genus *Potyvirus*, family *Potyviridae*) also encodes a Ham1 protein with an uncharacterized function (Knierim *et al*., 2016). EuRV, CBSV and UCBSV are part of a small number of viruses which are able to infect plants in the Euphorbiaceae family, and so perhaps Ham1 proteins are a euphorbia host adaptation (Monger *et al*., 2010).

## Differences Between CBSVs Infections and Genome Sequences

In 2010, CBSV was found in infected cassava samples from Mozambique and Tanzania and UCBSV in Kenya, Uganda, Malawi and northwestern Tanzania (Winter *et al*., 2010). However, recent phylogenetic analysis of whole genome sequences has revealed that the viral species are not limited to agro‐ecological zones, and that there may be three separate species within the UCBSV clade (Ndunguru *et al*., 2015).

CBSV and UCBSV produce distinctly different symptoms on cassava and indicator hosts. CBSV causes more severe root necrosis and feathery chlorosis along vein margins, which develops into chlorotic blotches, whereas UCBSV causes circular chlorotic blotches between veins in cassava (Mohammed *et al*., 2012; Nichols, 1950; Winter *et al*., 2010). CBSV tends to accumulate to higher titres than UCBSV in cassava (Kaweesi *et al*., 2014) and indicator plants (Mohammed *et al*., 2012; Ogwok *et al*., 2014).

Sequence differences between CBSV and UCBSV genomes should explain differences in symptom severity, viral load and host interactions observed between the two viral species. Key areas of CBSV and UCBSV genomes show relatively high levels of divergence, including the P1 and Ham1 regions, with only 59% and 47% amino acid identity, respectively (Winter *et al*., 2010). One suggestion for the low level of Ham1 sequence similarity is that Ham1 genes may have been acquired separately by CBSV and UCBSV from a eukaryotic host (Monger *et al*., 2010). Alternatively, CBSV and UCBSV Ham1 sequences may have been derived from a common ancestor, which may have diverged as a result of differential selection pressures on the genome sequences of the two species (Monger *et al*., 2010).

## Evolution of CBSVs

Statistical analysis of CBSV and UCBSV genomes using the empirical Bayes approach has predicted amino acid sites in UCBSV and CBSV coat protein and UCBSV Ham1 sequences, which appear to have been under positive selection (Mbanzibwa *et al*., 2011a). It is possible that positive selection at these different amino acid positions may have enabled the adaptive evolution of the two viral species (Mbanzibwa *et al*., 2011a). Recent whole genome sequence analysis has revealed that there is a higher diversity of CBSV isolates relative to UCBSV (Alicai *et al*., 2016). This diversity may have enabled CBSV to rapidly adapt to overcome host resistance mechanisms, which breeders have been selecting for (Alicai *et al*., 2016).

Whole genome analysis has also identified putative homologous recombination sites within the genomes of CBSV and UCBSV isolates (Ndunguru *et al*., 2015). To date, there has been no evidence for recombination between CBSV and UCBSV isolates (Mbanzibwa *et al*., 2011a; Ndunguru *et al*., 2015). However, the analysis of more CBSV and UCBSV genome sequences should provide insights into the importance of recombination in CBSD viral evolution.

## Potential Interactions Between CBSVs

There is potential for CBSV and UCBSV isolates to interact as RT‐PCR has revealed that mixed infections are common, making up 34%–50% of tested infections in Kenya (Kathurima *et al*., 2016), Tanzania (Mbanzibwa *et al*., 2011b) and Uganda (Ogwok *et al*., 2014). The potential interactions between the two viral species are not currently understood. Two of the CMD causal viruses, *African cassava mosaic virus* (ACMV) and *East African cassava mosaic virus* (EACMV), have been shown to interact synergistically, leading to increased viral titres (Vanitharani *et al*., 2004). It is therefore possible that similar synergistic interactions occur between CBSD viral isolates.

## Breeding for CBSD Resistance

To date, there is no cassava cultivar with a high level of CBSD resistance available to farmers (Abaca *et al*., 2013). Breeding cassava is notoriously difficult because of the high heterozygosity and a challenging cross‐pollination process (Ceballos *et al*., 2012). Breeding is further complicated by cultivars showing variation in CBSD resistance across different environments, which necessitates the testing of cultivars in different agro‐ecological zones to ensure that their resistance is stable (Tumuhimbise *et al*., 2014).

Breeders and farmers across Tanzania, Kenya, Uganda and Malawi have been selecting cultivars which strongly express foliar symptoms, but develop low levels of storage root necrosis (Hillocks *et al*., 2016). Twenty‐five best‐bet clones from five countries across East and southern Africa were selected, virus‐cleaned, shared and regionally evaluated across diverse environments for sources of CBSD and CMD resistance under the 5CP project (IITA, 2014b). Breeding efforts also include a 7‐year evaluation process of Tanzanian and Ugandan germplasm, whereby extensive intraspecific hybridizations have generated tolerant clones which develop relatively low levels of root necrosis of 12%, compared with >80% in sensitive cultivars (Kawuki *et al*., 2016).

Although tolerant cultivars develop reduced symptoms, they remain susceptible to CBSD viruses and thereby their adoption does not remove viral inocula from the field. Therefore, considerable efforts have been made to screen and breed cassava cultivars for CBSD resistance, which are able to restrict CBSD viral replication and/or movement. Promisingly, protoplast studies have recently shown that the elite breeding line KBH2006/18 can inhibit CBSD viral replication, which offers exciting opportunities to characterize resistance and resistance‐breaking viral virulence factors (Anjanappa *et al*., 2016).

### Responses of different cassava cultivars to cbsd

Cassava cultivars respond very differently to infection by CBSVs; they produce a range of symptoms and are associated with varying viral loads at different time points of infection (Kaweesi *et al*., 2014). Sensitive cultivars show severe shoot and root symptoms, whereas cultivars with higher tolerance tend to express foliar symptoms, but usually lack or exhibit mild root necrosis (Hillocks and Jennings, 2003). Cultivars such as NASE 3 show high levels of resistance to UCBSV infection, but remain susceptible to CBSV (Ogwok *et al*., 2016). It has been shown that cultivars, such as ‘Namikonga’, support lower viral titres than susceptible cultivars, such as Albert (Maruthi *et al*., 2014). However, symptom severity is not always correlated with viral load, as the cultivar NASE 1 supports a relatively high viral load, but produces no foliar or root necrosis symptoms, whereas the cultivar NASE 14 supports a low viral load, but expresses severe root necrosis (Kaweesi *et al*., 2014).

This disparity between viral titres and symptom development has necessitated the use of viral load quantification during breeding to identify and select cultivars which support low CBSD viral titres. Until recently, the quantification of CBSD viruses in cassava was based on quantitative RT‐PCR, which measures the abundance of viral transcripts relative to the abundance of plant reference gene transcripts (Abarshi *et al*., 2012; Kaweesi *et al*., 2014; Moreno *et al*., 2011; Ogwok *et al*., 2014). However, the expression of plant reference genes can vary in different plant tissues, under varying developmental and environmental conditions (Brunner *et al*., 2004) and during viral infection (Liu *et al*., 2012). To overcome this, Shirima *et al*. (2017) have recently adapted the quantitative RT‐PCR technique to enable the absolute quantification of CBSV mRNA without normalization to plant reference genes. The higher levels of accuracy offered by this technique should be valuable in breeding efforts to generate cassava cultivars which support very low CBSD viral loads.

### Identification of cbsd tolerance markers in cassava genomes

Despite the importance of cassava in developing countries, it has received relatively little scientific attention when compared with maize, rice and wheat (Varshney *et al*., 2012). Genomic studies of cassava are now enabling the identification of genetic markers associated with tolerance within the genomes of tolerant cultivars. In 2009, the first cassava genome assembly and annotation was publicly released (Prochnik *et al*., 2012). Since then, a large linkage map has been built using simple sequence repeats (SSRs) and single nucleotide polymorphisms (SNPs) to identify quantitative trait markers associated with CBSD tolerance across diverse African farmer‐preferred cultivars (Patil *et al*., 2015; Prochnik *et al*., 2012). This has revealed a number of putative CBSD tolerance alleles across different chromosomes in different cassava genotypes (Abaca *et al*., 2013; Nzuki *et al*., 2017). If validated, these alleles will be useful as markers in marker‐assisted breeding and could be combined into cultivars for effective and durable CBSD tolerance (Pariyo *et al*., 2013).

### Transcriptional responses to cbsd viruses in different cassava cultivars

To date, very little is known about the function of these putative CBSD tolerance alleles. RNA sequencing analysis of transcripts, which are overexpressed during CBSD infection of the tolerant cultivar ‘Namikonga’, has implicated NAC transcription factors, as well as genes involved in jasmonic acid hormone signalling and the biosynthesis of phenylpropanoid, terpenoid and steroid secondary metabolites (Maruthi *et al*., 2014). In other plants, jasmonic acid and secondary metabolites are linked to abiotic and biotic stress responses (Izbiańska *et al*., 2014; Petrussa *et al*., 2013; Wasternack and Hause, 2013).

Transcriptional studies are also helping to gain an understanding of the mechanisms behind these different interactions between cassava cultivars and different CBSD viruses. Ogwok *et al*. (2016) have demonstrated recently that Dicer‐like proteins 2 and 4 (DCL2 and DCL4) and Argonaute 2 (AGO2) are differentially expressed during CBSV and UCBSV infections in different cassava cultivars. DCL and AGO proteins are integral to the plant antiviral defence mechanism of silencing viral RNA (Llave, 2010). Further studies are required to gain a fuller understanding of how the genes involved in host silencing of viral RNA are differentially expressed in different cultivars in response to different CBSVs.

Transcriptome analysis has also revealed that β‐1,3‐glucanase, which is involved in callose degradation at plasmodesmata, is up‐regulated during CBSD infection of the susceptible cultivar 60444, but not in the elite breeding line KBH2006/18, which shows relatively high levels of CBSD resistance (Anjanappa *et al*., 2017). The degradation of callose at plasmodesmata has been shown previously to promote viral movement (Zavaliev *et al*., 2011). Anjanappa *et al*. (2017) have suggested that enhanced callose degradation at plasmodesmata during CBSD infection of 60444 may promote viral movement, whereas the greater amount of callose present at plasmodesmata during KBH2006/18 infection is sufficient to limit systemic viral movement and thereby restrict infection.

### CBSD resistance through genetic transformation

There have been promising attempts to introduce CBSD resistance into cassava through genetic engineering. The mechanism utilized involves the transgenic expression of inverted repeat CBSD viral sequences to trigger post‐transcriptional gene silencing (PTGS) of the corresponding sequences during infection, and hence confer viral resistance to the plant (Patil *et al*., 2011). The approach was successful in *N. benthamiana*; transgenic expression of UCBSV coat protein hairpin constructs resulted in high levels of resistance to six diverse CBSV and UCBSV isolates (Patil *et al*., 2011). The same construct was expressed in cassava and conferred resistance to CBSV and UCBSV under field conditions with high disease pressure (Ogwok *et al*., 2012; Yadav *et al*., 2011). Vegetative stem cuttings taken from transgenic plants retained CBSD resistance, enabling their use in vegetative propagation (Odipio *et al*., 2013).

To ensure that transgenic plants are resistant to both CBSV and UCBSV, the cultivar TME 204 was transformed with a construct (p5001) containing fused tandem repeat coat protein sequences from both CBSV and UCBSV to produce the transgenic line: TME 204 p5001 (Beyene *et al*., 2017). This transgenic line was resistant to CBSD when graft challenged (Beyene *et al*., 2017) and grown within confined field trials across different agro‐ecological locations in Uganda and Kenya, where plants were exposed to a range of both CBSV and UCBSV isolates over multiple vegetative propagation cycles (Wagaba *et al*., 2017).

It is vitally important that improved cultivars are resistant to both CBSD and CMD. Transgenic CBSD resistance was conferred to cultivars TME 7 and TME 204, which are naturally CMD resistant as a result of the presence of the single dominant CMD2 resistance locus (Beyene *et al*., 2016; Vanderschuren *et al*., 2012). Critically, however, these TME cultivars lost their CMD2 resistance through an unknown mechanism during somatic embryogenesis (Beyene *et al*., 2016). Work is ongoing to cross the CBSD‐resistant transgenic line TME 204 p5001 with a wild‐type CMD2 type cultivar to combine durable CBSD and CMD resistance into a single cultivar (Beyene *et al*., 2017).

Once biosafety issues have been addressed, the potential benefits of genetically modified cassava to smallholder famers will be substantial. It has been estimated that the net value of the release of CBSD‐resistant cultivars will be US$436 million for western Kenya and US$790 million for Uganda over a 35‐year period starting in 2025 (Taylor *et al*., 2016). The Virus Resistant Cassava for Africa (VIRCA Plus) project is working to deliver CMD‐ and CBSD‐resistant cassava cultivars to smallholder farmers in Uganda and Kenya, and so improve their livelihoods and food security (Taylor *et al*., 2016).

## Distribution of Certified Virus‐clean Planting Material

The lack of cultivars highly resistant to CBSD makes the existence of a clean seed system critical for the effective management of CBSD. Clean cassava seed systems are non‐existent in most eastern Africa countries where CBSD is a problem. The Great Lakes Cassava Initiative was launched in 2008 with an overall goal to distribute certified virus‐clean CBSD‐tolerant cultivars to 1.15 million farmers across six East and Central African countries over a 4‐year period (Catholic Relief Services, 2010). As tolerant cultivars still retain viruses within their stems, planting material must be subjected to a cleaning process and highly sensitive diagnostic testing before it can be multiplied and supplied to farmers. This should reduce disease pressure in affected areas, as at least initially the majority of crops will be disease free (Mwangangi, 2014). The production of certified virus‐clean cassava germplasm is particularly important during the transportation of vegetative planting material because of the risks posed by CBSD to cassava‐growing areas which are currently unaffected (Legg *et al*., 2011). The cleaning process involves culturing meristem tissue *in vitro*, and subjecting it to thermo‐ and/or chemotherapy, which inactivates viruses and prevents viral replication or movement within tissues.

Mathematical modelling has shown that, in order for the clean seed system to be sustainable, multiplication sites should only be set up in areas with low disease pressure and low vector population density (McQuaid *et al*., 2015). Modelling has also shown that, to reduce CBSD dispersal and increase cassava yields, virus‐free planting material should be distributed to a number of different growers across a widespread area with restricted trade (McQuaid *et al*., 2017). Once certified virus‐clean material has been distributed, farmers must also be thoroughly trained in the identification of disease symptoms to enable sufficient roguing to further reduce CBSD spread (Legg *et al*., 2017; McQuaid *et al*., 2015). Cassava clean seed system projects have recently been piloted in Uganda and Tanzania. (Legg *et al*., 2017; McQuaid *et al*., 2015).

## CBSVs Diagnostics

As many CBSD‐infected plants remain symptomless, highly sensitive diagnostic techniques are required in the production and transportation of material (Abarshi *et al*., 2010). There have been several important advancements in cassava disease diagnostic techniques, including the optimization of RT‐PCR to enable reliable simultaneous detection of CBSV and UCBSV (Mbanzibwa *et al*., 2011b), as well as cassava mosaic begomoviruses, in a single multiplex RT‐PCR (Abarshi *et al*., 2012). Next‐generation high‐throughput sequencing (NGS) has been used to screen large numbers of plants for the presence of CBSVs to ensure that they are virus free before dissemination as planting material. Adams *et al*. (2013) demonstrated that, with NGS, it was possible to detect 1% of infected plants out of a total of 300 plants with 95% probability. Although useful tools, to date, many of these techniques are relatively resource intensive, and so it is vitally important that affordable diagnostic tools are available in African countries to enable sensitive CBSD detection locally, even in cassava fields. One promising technique is reverse transcription loop‐mediated isothermal amplification (RT‐LAMP), which is able to detect and differentiate the presence of CBSV and UCBSV with lower consumables, resources and instrument costs than RT‐PCR (Tomlinson *et al*., 2013).

## Conclusions

In the past 20 years, CBSD has become a major cause of food insecurity across East and Central Africa, and only since its recent geographical expansion has the disease received the scientific interest it deserves. Once the CBSD pandemic unravelled, it was largely too late to restrict the disease to limited outbreak areas. Lessons must be learnt from this to prevent similar disease outbreaks in the future. Critically, scientific interest should be applied to the prediction and prevention of future outbreaks before they are able to emerge and cause devastating yield losses across large areas.

In terms of understanding CBSD, recent studies have begun to show that CBSVs are diverse and that CBSV has a high evolutionary capacity (Alicai *et al*., 2016). Many control efforts are being aided by advancing molecular techniques, including marker‐assisted breeding, development of genetically modified resistant lines, provision of certified virus‐clean planting material and the use of sensitive diagnostics. Despite this progress, there are still many areas of CBSD biology and epidemiology which remain poorly understood and offer opportunities to further understand and control the disease.

## Future Prospects

### Understanding key drivers in CBSD epidemiology

Relatively little is known about the complex interactions between viral variants, vectors, cassava cultivars and environmental conditions, and how they may influence the spread of CBSD. Therefore CBSD incidence, prevalence and whitefly populations in farmers' fields need to be regularly monitored in major cassava‐producing areas to track periodic changes in the general status of the disease in affected countries and those at risk. Where control interventions are deployed, they should be evaluated for their impact in the control of CBSD. The availability of this information is required for the development of predictive models that will provide an evidence base for disease control decisions and resource allocation. The effectiveness of CBSD control strategies is also heavily dependent on the level of farmer engagement and awareness. In Uganda, extension work includes efforts to raise farmer awareness of CBSD and to deliver information on its management (Kumakech *et al*., 2013).

### Gaining insights into viral populations

We currently know very little about viral populations within wild hosts, which may serve as important sources of viral inoculum and enable the evolution of CBSD and other emerging viral diseases. Next‐generation deep sequencing can be used to detect viral populations about which very little sequence information is known (Prabha *et al*., 2013). It would be fascinating to apply this to cassava and to characterize viral populations within CBSD‐infected cassava and wild hosts surrounding cassava crops. This could shed light on viral evolution and the contribution of wild hosts to epidemiology. It may also help to identify potential unknown viral diseases, against which pre‐emptive control could be taken in anticipation of emerging diseases (Newbery *et al*., 2016).

### Measures to restrict CBSD spread into unaffected areas

To date, CBSD viruses are only found in East and Central Africa. However, CBSD distribution could increase should infected material be transported to other cassava‐growing areas of Africa, Latin America and Asia, which would result in huge economic losses and food insecurity (Legg *et al*., 2014). Therefore, the movement of cassava material from CBSD‐affected countries should be subject to strict quarantine measures to ensure that planting material is virus free before transportation. Such measures will facilitate the movement of superior cultivars for production or breeding purposes.

### Utilizing diverse cultivars for genomic resources

It is important to continue to maintain and investigate diverse cassava germplasm from across Africa and Latin America and their wild relatives for potential sources of disease resistance and other beneficial agronomic traits (Turyagyenda *et al*., 2012). This will enable farmers to adapt to changing environmental, socio‐cultural and market conditions (Pautasso *et al*., 2013).

### Surveillance of viral diseases

To target control efforts, it is vitally important to accurately survey viral disease distribution. The IITA has recently launched the Cassava Disease Surveillance Platform in Nigeria, which offers opportunities for cassava breeders and extension workers to upload images of plants suspected to be infected with CBSD and other diseases. The images are analysed by a team of experts to enable rapid diagnosis and coordination of emergency control responses (IITA, 2016). Similarly, the West African Virus Epidemiology Project launched in 2015 aims to use field surveys to gain a clear understanding of the viruses which affect cassava in West Africa to predict viral emergence and inform policy decisions. Structured surveys under the Cassava Virus Diagnostics Project in eastern and southern Africa are tracking area‐wide changes in cassava viral diseases over time. This will provide the basis for disease control intervention decision‐making and impact assessment.

### Predicted effects of climate change on cassava production

Cassava demonstrates relatively high levels of resilience to temperature and rainfall fluctuations predicted in climate change models (El‐Sharkawy, 2004). A model based on temperature and rainfall projections across Africa has predicted that, compared with other staple food crops, overall cassava is the least likely to be adversely affected by climate change (Jarvis *et al*., 2012). This makes cassava an attractive food security crop for climate change adaptation in Africa. However, climate change is also predicted to affect the distribution and abundance of cassava pests and diseases, including *B. tabaci* (Jarvis *et al*., 2012). Recent ecological niche modelling has predicted that, with climate change, the potential distribution of CBSD‐ and CMD‐carrying *B. tabaci* will spread over West, Central and southwestern coastal Africa where cassava production is high and CBSD is currently absent (Herrera Campo *et al*., 2011). Therefore, the monitoring and control of *B. tabaci* populations are major priorities. The deep sequencing technique could be extended to *B. tabaci*, enabling the mapping of the most active and abundant viral species carried by *B. tabaci* populations across different agricultural regions (Ng *et al*., 2011).

### Understanding CBSVs infection mechanisms and virulence determinants

Despite the increasing number of sequenced CBSV genomes, very little is known about the virulence determinants within CBSV and UCBSV genomes responsible for key functions during infection, and their effect on disease symptomatology. To date, only the silencing suppression activity of the UCBSV P1 protein has been characterized (Mbanzibwa *et al*., 2009a). The construction of infectious clones will enable the targeted mutagenesis of key viral sequences to identify the functions of viral proteins and the host proteins with which they interact, which should serve as potential targets to restrict viral infection. Current work to develop and manipulate CBSV infectious clones is ongoing at various institutions.

### Collaborative sharing of information and resources

There are many opportunities to exploit the recent progress made in understanding CBSD through progress in cassava, viral and vector research. There is a need for this research to be integrated into a central, easily accessible platform (Ayling *et al*., 2012). This will require experts across diverse backgrounds and countries to openly communicate, engage, share data and collaborate through networks, such the Global Cassava Partnership for the 21st Century. Such partnerships should help to generate solutions to control CBSD and enable cassava to fulfil its potential of feeding billions of people by 2050 (Legg *et al*., 2014).

## Conflict of Interest

The authors declare that they have no conflicts of interest with the contents of this article.
